# The change in motivating factors influencing commencement, adherence and retention to a supervised resistance training programme in previously sedentary post-menopausal women: a prospective cohort study

**DOI:** 10.1186/s12889-015-1543-6

**Published:** 2015-03-12

**Authors:** Janet Erica Viljoen, Candice Jo-Anne Christie

**Affiliations:** Department of Human Kinetics and Ergonomics, Rhodes University, African Street, PO Box 94, Grahamstown, 6140 South Africa

**Keywords:** Physical activity, Postmenopause, Adherence, Retention, Strength training

## Abstract

**Background:**

Understanding motivators for exercise participation in post-menopausal women may impact retention to exercise programmes and inform intervention trial designs. The purpose of this investigation was to assess self-reported motivational factors influencing adherence and retention to a 24-week progressive resistance training programme.

**Methods:**

Post-menopausal females (n = 34) were passively recruited to undertake a 24-week progressive resistance training protocol, in small-group sessions, on three non-consecutive days of the week. Attendance was recorded by the researcher. Qualitative reports were sourced from the sample for four phases of the study: pre-study (prior to week 1), recruitment (week 1), during study (weeks 2 – 24), and post-intervention (beyond week 24). Responses were categorised according to ten descriptors: specific health index improvement, education, flexibility of time, social contact, conscience (loyalty to the researcher), wellness, weight management, organisation parameters (pertaining to the study programme) and enjoyment of the exercises.

**Results:**

Of the initial sample, 76.5% (n = 26) met the specified ≥80% attendance criterion. The primary findings were that motivation to volunteer for the study was driven by a perceived need for a structured exercise programme (50% of respondents). A commitment to the researcher was the primary motivator for continued adherence to the study for 50% of participants. Social contact with other participants was cited by 60% of the sample as the primary reason for adherence for the full duration of 24 weeks. A desire to maintain the “wellness” derived from the programme was cited by 60% as a reason for continuing an exercise routine post-study.

**Conclusion:**

This study identified that routine and supervision initially attract women to exercise programmes, while social cohesion of the group setting contributes to retention over time. Understanding the changing nature of motivating factors may contribute to better overall adherence and retention to exercise programmes and interventions.

## Background

Interventions to treat chronic conditions may take the form of drug or lifestyle interventions. The success of either approach is determinant on long-term adherence by the individual. There is an implicit understanding that should a desirable effect be found that adherence to the treatment in the long-term is critical for beneficence. Despite this, participant attrition remains a pivotal hurdle for researchers embarking on studies of this nature: not only is the veracity of the study called into question, but the individuals who stood to benefit willingly forego health improvements [1,2].

Motivation to adhere to exercise programmes appears poor in female populations compared to male samples, and in older age groups compared to younger individuals, despite the fact that the benefits of exercise are well known [3]. Adherence is reportedly especially problematic in cohorts of post-menopausal women [3]. An understanding of the factors that serve to motivate individuals is therefore critical not only to improving retention rates during the course of longitudinal trials but also for permanent lifestyle change behaviour [4]. Previous studies have identified the role of the setting of the intervention as critical to adherence and retention rates [5,6]. Supervised group settings produce higher retention than individual or home-based programmes [5]. The “Sedentary women exercise adherence trial” (SWEAT) study found that the type of activity did not affect retention in a comparison of two different modes of aerobic activity, and concurred that supervised settings were more effective in retaining participants [7]. Other investigators report that the rapport between the research team and the participants is critical in preventing attrition, and that high turnover of research team staff is a negative influence on the cohort [8]. Practical barriers to participation include time, transport, costs involved and procedures [4].

If exercise is to be considered an important treatment mechanism in the context of burgeoning lifestyle related diseases, an understanding of the characteristics of individuals who do not adhere is useful. Drop-outs from longitudinal studies are characterised as older, female individuals with a higher degree of medical burden. Consequently the health status of these individuals is typically worse than those who remain in the study cohort for the stipulated period of the intervention [1].

Most studies have employed aerobic endurance training as the exercise modality. Progressive resistance training (PRT) has, however, been recommended by the American College of Sports Medicine (ACSM) as a necessary adjunct training modality to aerobic endurance training, specifically in older individuals prone to the loss of lean body mass through the aging process, to be undertaken on at least two days of the week (with the proviso that exercise is undertaken on at least five days of the week, for at least 30 minutes per session).

The purpose of this study was to assess self-reported motivational factors influencing commencement, adherence and retention to a long-term, supervised PRT programme in a group of post-menopausal females. A further aim was to assess motivators for continued exercise participation after 12 weeks following completion of the trial.

## Methods

This study was designed as a prospective cohort investigation, and as such no control group was utilised.

### Study overview and organization

The study formed part of a larger investigation which explored the impact of a supervised PRT intervention programme on cardiovascular risk factors, specifically abnormal blood lipid profiles, in a group of previously sedentary, post-menopausal females [9]. The main focus of this sub-study was to prospectively determine factors which influenced commencement, adherence and retention to the exercise programme.

### Participants

Previously sedentary, South African, post-menopausal females (n = 34) aged between 50 and 75 years (mean 58.94 ± 14.22 years, range 50 – 71 years, median age 55 years), were encouraged to participate in a 24 week PRT programme.

Recruitment took place by means of advertisements in local media, local pharmaceutical outlets and medical practitioners’ rooms. Individual declarations that regular menses had ceased at least 12 months prior were obtained from the individuals. An information briefing was held for all interested individuals prior to commitment to the study.

### Inclusion criteria

The following criteria were considered when recruiting individuals for participation for the larger study of which this investigation formed a part [9].

*Female:* it is now accepted that cardiovascular disease is a primary cause of mortality amongst females, particular those post-menopause. In addition, attention to female samples is required, as only ~20% of trial participants to date have been female [10].

*Age 50 to 75 years*: an older, female sample was required for this study. While the range selected is broad and may incur age-related limitations, the need to recruit a large enough cohort determined this criterion. Most cardiac changes have been evaluated in men. Once menopause has passed, it is acknowledged that women are at greater risk for cardiovascular and related complications [11-13]. The mean age at which the menopause occurs is 50 years of age, and the majority of women are post-menopause by the age of 52 years [13].

*Post-menopausal:* it is known that prior to the menopause females are at lower risk for CVD than male counterparts. Once ovarian function has ceased this landscape changes dramatically. As CVD is the primary killer of females post-menses, attention to this sample is required [14-17].

*Healthy:* self-reports of no heart, lung, liver or kidney disease, as medication for certain conditions would mask the effects of the exercise intervention [18-21].

*Able to undertake exercise*: all participants were of sound musculo-skeletal health. As is recommended for women over the age of 50 years, all candidates were examined by a medical practitioner prior to undertaking the exercise intervention [11].

*Sedentary:* defined as less than 30 minutes of structured or continuous activity on fewer than three days of the week. The ACSM defines the minimum volume of activity required for health benefits in adults as 30 minutes of moderate activity on at least five days of the week, updated from the previous recommendation of three days per week [11]. Self-reports were corroborated by assessing the baseline ‘Community health activities model program for seniors’ (CHAMPS) questionnaire, and were monitored throughout via repeat submissions thereof.

*Non-smokers:* participants were all non-smokers as smoking is known to affect not only indices of health but may be a contributing factor when examining motivation to exercise. If they had previously smoked, they had to have given up the habit at least 12 months prior to recruitment [22-25].

### Pre-screening

All participants signed written consent following extensive information dissemination, and the protocol was approved by the Rhodes University Ethical Standards Committee for research involving human participants according to the Declaration of Helsinki (1975, revised in 2000). Once participants had consented, pre-screening was undertaken in the week prior to commencement of PRT. This took place with the assistance of a medical practitioner on two afternoons of the week in a clinical laboratory. All were screened using a standard physical examination protocol that included a submaximal electrocardiogram (ECG) test and an oral glucose tolerance test as described previously [9]. In addition, no individuals had any heart, lung, liver or kidney disease that precluded moderate intensity exercise as determined by self-reports and confirmed via medical screening.

### Baseline measures

The measurements obtained at baseline and every four weeks thereafter have been reported elsewhere [9].

### Exercise programme

Exercise sessions commenced thereafter, supervised at all times by female postgraduate students and the principal researcher in a private laboratory. Sessions were timetabled according to the time preference of the volunteers, and were held at 6 am, 1 pm, 4 pm and 5 pm on four days of the week (individuals only had to attend on three days, the additional day was a “catch up” session available for participants that were unable to attend on another day). Every four weeks the initial measurements (described above) were repeated at the same time of day as at baseline, and by the principal researcher. It must be noted that there was no dietary manipulation included in this investigation. Participants were requested to continue habitual dietary intake and were advised that body mass loss >5% of baseline body mass would result in exclusion from the data set.

#### Exercise protocol

A 24-week PRT occurred on three non-consecutive days of the week. Intensity was initially set at 50% of the pre-determined 1-repetition maximum (1RM) test of muscular strength, and was increased every four weeks to a maximum of 80% of the 1RM. A battery of ten exercises was completed during each 45 minute training session, which included a five minute aerobic warm-up undertaken on a cycle ergometer (Cateye Ergociser EC1600, Japan).

Intensity rose by 10% every four weeks and difficulty was also progressed as muscular strength and skill developed over time, and in order to fulfil the requirement that the programme be a “progressive, resistance” training programme. The exercises included were: chest press (initially on a machine and then using free weights); leg extensions (machine based); abdominal crunches (using a weight stack machine and progressing to a Swiss ball); travelling lunges (initially unweighted and progressing to the addition of hand-held weights); back extension (using a weight stack); hip extension (using a weight-stack machine); shoulder pull-down (machine-based) and “lat” pull-down (“latissimus dorsi” pulldown using weight stack machine, initially seated on a solid surface and later progressing to sitting on a 65 cm diameter exercise ball). All weight-stack machines were commercially available gymnasium equipment (Challenger series by Zest Fitness®, Cape Town, South Africa).

#### Attendance criteria and data

The volunteers were advised that a minimum attendance criterion of 80% was required for inclusion into the data set. The rationale for this was explained, and a verbal commitment requested prior to signing consent. At each session an “attendance register” kept in the laboratory was completed by the supervising researcher with the knowledge of the attendees. Frequent communication between the principal researcher and the volunteers was encouraged and when this did not occur ‘face-to-face’ the primary means of contact was via email. Thus, explanations for absences as well as notifications of illness were provided voluntarily and recorded.

#### Self reports

Immediately following the intervention all participants were canvassed indirectly via email for self-reports on motivating factors that may have contributed to the successful adherence to the study. The responses were guided using a four-phase distinction, namely: phase one – motivation to contact the researcher to sign up for the programme; phase two – motivation to commit to the study following screening and information processes; phase three – motivation to continue with the programme during the 24-week period; and phase four – motivation to continue exercise post-intervention without the supervision or the structure of the study in place. Twelve weeks post-study all recruits were contacted again and asked to report on whether they had continued regular exercise beyond the conclusion of the intervention. In addition, participants were asked to report on why they had, or had not managed to continue routine activity.

Responses were further categorised into ‘descriptors’ for ease of data management. Similar responses were categorised together but no response was erased nor were responses changed to fit existing categories. Nine ‘descriptors’ emerged: health indices (the desire to improve known or suspected health concerns, such as abnormal lipid profiles or hypertension); structured routine (the acknowledgement that an external ‘timetable’ or ‘appointment’ would aid in adherence); education (the express wish to learn more about certain health parameters); time flexibility (the attraction of being able to choose session times according to lifestyle demands rather than having to attend one specific timeslot); social contact (interaction with individuals of similar age and status as well as a small group context which facilitated social interaction during the exercise sessions); conscience (the commitment to the principal researcher and loyalty to this commitment); wellness (an understanding of the role of habitual exercise in general health and wellbeing, without necessarily requiring a specific element to improve); weight management (for many females there is a desire to lower body mass, and for this post-menopausal sample the need to stabilise body mass and prevent further gains was important); and finally enjoyment of exercise (actual enjoyment of undertaking the battery of exercises and the act of exercising).

Respondents were permitted to refer to more than one parameter, in order of preference, where necessary.

#### Statistical analysis

Descriptive statistics were obtained using Statistica (version 10) software (www.statsoft.com).

## Results

Thirty five recruits underwent screening, during which one individual was rejected following an abnormal post-challenge blood glucose reading (an indicator of pre-diabetes). Four respondents were non-Caucasian. The study commenced with thirty four participants. The retention rate was n = 26 while the attrition rate was n = 8. The characteristics of the sample are presented in Table 1.

**Table 1:** **Sample characteristics before, and on completion of, 24 week intervention**

	**Age**	**Mass**	**Body mass index**	**Waist circumference**
	**(years)**	**(kg)**	**(kg.m** ^**−2**^ **)**	**(cm)**
**Baseline**	58.9 ± 14.2	79.1 ± 17.4	29.8 ± 6.8	95.0 ± 15.0
**24 Weeks**	58.9 ± 14.2	78.3 ± 16.8	29.5 ± 6.5	90.2 ± 13.2

### Motivating factors by study phase

Each respondent provided one to three motivating factors per phase, and these have been summarised accordingly.

#### Phase 1: Initial contact

Communication with the researcher following advertising of the study was driven by the desire for routine for 60% of respondents (Table 2). In addition 50% reported that further knowledge about the health problem to be researched during this intervention, in this case abnormal blood lipid profiles, was important to them. The interest in a social setting as well as a recognition that the advertised research should be supported in a relatively small community were each responsible for 10% (a total of 20%) of respondents contacting the researcher for further information.

**Table 2:** **Most frequently cited motivators at each phase of the study**

**Motivator**	**Initial contact**	**Informed consent**	**Retention during study**	**Continuation post study**
	**(%)**	**(%)**	**(%)**	**(%)**
Routine	**60** ^1^			
Cholesterol	50	**30**		
Social Cohesion	10		**60**	
Conscience/Loyalty	10		50	
Education		20		
Time Flexibility		20		
Wellness			30	**60**
Weight Management				20
Enjoyment				10

^1^Factors in boldface were primary motivators at each stage, highlighting the shift in motivation.

#### Phase 2: Recruitment and informed consent

Once respondents had attended the introductory information briefings which were facilitated by the principal researcher in a social setting, 30% were motivated to participate based on new knowledge about abnormal lipid profiles and the risk of cardiovascular disease in post-menopausal female samples (Table 2).

A further 20% recognised that education about health, and in particular cardiovascular risk, was important and decided to commit to the study as a means of furthering this knowledge. Advertised flexibility of session times became a facilitator to commitment for 20% of the recruits at this stage – despite no session times having been confirmed.

#### Phase 3: Retention during study

Commencement of the 24-week programme was a considered commitment by each participant. Consent was signed following full briefing and pre-screening, in full knowledge and understanding of the commitment to the study, as well as the risks and potential benefits. By this stage 50% of those participating were motivated to honour a commitment they viewed as having been made to the principal researcher – it was the relationship with this person rather than with the “study” that drove this motivation (Table 2).

The influence and emphasis on relationship dynamics are underscored as a further 60% of respondents cited the social group setting as the motivator that assured their continued adherence to the programme. Wellness, at this stage not directly related to cardiovascular risk profile but of a more ‘general’ nature, was the factor that influenced 30% to adhere to the exercise programme.

#### Phase 4: Continuation post-study

Following the official completion of the supervised exercise study duration, participants expressed an interest in continuing sessions. As part of the ethical obligation to the participants, advice was offered about other exercise programmes or facility options available in the vicinity. Twelve weeks post-intervention the participants were canvassed as to ongoing exercise and 60% confirmed continued habitual activity while 40% described ongoing exercise as “irregular” – fewer than three days of the week and irregularly so. Health problems and frequent travel were cited as hurdles to regular activity by 20% while the absence of social interaction had deterred a further 20% from continuing to exercise regularly. For those who had continued to exercise habitually (at least three days of the week or more) the motivator was “wellness” (60%) and the association between physical activity and feelings of wellbeing both physically and psycho-emotionally, followed by weight management (20%) and enjoyment of the activity (10%) (Table 2).

## Discussion

The time change course of motivating factors within a post-menopausal sample of South African middle class women were identified. Initial contact made with the researcher was driven by the appeal of a routine of exercise (60% of respondents), while retention to the study during the 24 week intervention was primarily motivated by the social cohesion of the small group setting (60% of respondents) followed closely by the relationship with the researcher (50% of respondents). Long term and continued adherence to exercise beyond the end point of the intervention was motivated by the desire to maintain a state of wellbeing (60% of respondents).

While barriers to exercise have been identified in older, female populations, the factors that influence adherence and retention to exercise programmes are less well understood [26]. As older individuals are found to be less likely to meet activity-related public health goals it is important for practitioners and health professionals to understand the factors that may prohibit previously sedentary individuals from adhering to long-term exercise plans [27]. This sample engaged women aged from 50 to 75 years, admittedly a broad age range, yet no correlation was identified between age and retention, indicating that prior information suggesting age is a barrier in itself to exercise may not hold true for all populations [27].

This study identified that the primary factors motivating post-menopausal participants to volunteer for the exercise programme was the promise of a supervised routine in a group setting (60% of respondents). This is similar to the findings of a study by McArthur and colleagues engaging “middle aged” women (age 40 – 62 years) [26]. It does however differ to previously published reviews on barriers to exercise in older populations which suggest that it is primarily health concerns, illness and time constraints that preclude regular activity in older populations, whereas these factors were not mentioned as barriers by the current sample [26,28,29]. Further limitations that have been posited include socio-economic concerns (such as the cost of joining a health facility) and the presence of symptoms of chronic diseases, but neither of these featured in the current sample [30,31]. Of importance in this regard is that the sessions on offer were free of charge, thus negating this barrier altogether. An important consideration for practitioners would be overcoming the financial cost barrier until such time as the individual recognises the value of the outlay and no longer recognises this as a barrier. The characteristics of individuals who voluntarily sign up for exercise interventions of this nature may be different to those who do not, however, and this must be considered when evaluating these findings as these individuals may present with higher levels of intrinsic motivation [29].

It was also clear that the motivating factors do change over time, and that ongoing adherence to the 24 week protocol was driven by commitment to the researcher and the study (50%) followed by the social cohesion of the small group setting (60%). The motivating element of group cohesion has been noted previously as significant for both short and long term adherence [31,32].

Of importance is the definition of “adherence”, and the fact that completion of an exercise programme does not automatically infer accrued health benefit [29]. In order to truly improve health status the duration of the exercise programme as well as the intensity of exercises prescribed must be completed. In the case of this sample, 76.5% of the initial sample attended more than 80% of the sessions offered. Previous literature has identified that an adherence rate of 80 – 85% is required for benefit to health [31]. While our study did not include a comparative group, studies that examined both PRT and aerobic endurance training have found that adherence to resistance training is better (95.4% in the case of pre-menopausal females, and 56.2% in ‘older’ females) compared to the aerobic training regime (65.4% for pre-menopausal women and 49.7% in the case of women of a mean age of 70.5 years) [30,31]. This supports the use of PRT as a preferred exercise modality for middle-aged and post-menopausal women.

As was found in previous research, poor perceptions of body image and the clear indication that the commercial gym environment is a deterrant to post-menopausal women was noted in this sample [30]. Body image is intricately linked to perceived self-confidence, and adults with lower self-confidence are less likely to commence activity from a previously sedentary starting point [27]. The perception that the individual is inexperienced in the gym environment, sensitivity about weight and body shape, as well as the presence of symptoms of poor health contribute to preventing otherwise healthy adults from engaging in regular activity [27,30]. This understanding may assist those who are involved in encouraging regular participation in physical activity for health benefit to motivate individuals and retain them on structured programmes.

### Study limitations

Limitations of this study must be acknowledged. Information pertaining to motivating factors was obtained via self-report, but as was the case in a similar study the principal researcher did obtain regular quantitative data throughout the course of the six month trial [33]. Despite widespread advertising in local media, as well as dissemination of flyers at local pharmacies and medical practitioner’s consulting rooms, the study primarily attracted educated Caucasian females. This pattern was noted in a previous study of adherence and represents a limitation to the applicability of the findings of this research [33]. It has been noted by researchers engaged in the large-scale ‘Women’s Health Initiative’ (WHI) trial that cultural competency and sensitivity to diversity are important skills needed in research team staff members in order to overcome the ethnic representation gap [2]. It is acknowledged that in the unique context of semi-urban South Africa that culturally specific motivators would apply and that future research must include a greater number of members of diverse ethnic groups.

The information was also obtained without due attention to the level of support offered to the participants from family or community members. As was the case in prior studies, this lack of information was a drawback as critical insight into reasons for attrition could have been obtained [6]. These omissions to the data set must be considered in future research.

Determination of menopause status in this research relied on the participants’ honest reports. In future studies, tests of follicular stimulating hormone (FSH) and/or estradiol levels via venepuncture and blood analysis would provide an objective measure of hormone levels indicative of menopause status. This study lasted 24 weeks duration which is sufficient to be considered “long term”, however, a better analysis of “long term” adherence to an exercise regime may require observation over 12 months to two years. It is also noted that 60% of those who commence an exercise programme will drop out within the first 24 weeks, and so insight over this period of time is crucial to long-term retention [31]. Finally, the sample size is admittedly small and indeed limits the interpretation of the results.

### Implications

Practitioners, policy makers or researchers who wish to include an exercise intervention as part of a lifestyle behaviour modification programme may benefit from the insights presented in this paper. It is well known that poor adherence to lifestyle modification programmes, particularly those involving exercise, often leads to the failure of the programme in reaching its intended goals. Understanding the shifts in motivators as demonstrated in this post-menopausal female cohort may lead to better success in such community-based programmes.

The fact the motivators changed over time for the current sample is clear. This research indicates that first contact success may be based on the individuals’ desire to have a routine-driven appointment for exercise sessions, rather than a self-appointed, self-managed schedule. Once recruitment is underway, the participants are motivated by the thought of better understanding the particular health concern or lifestyle change in question. During the intervention, the cohesion afforded by a small group setting and by the leader of the programme was demonstrably pivotal in retaining the recruits on the programme. It is possible that this cohesion could be engendered by instructors and trainers, but it may be that another form of relationship must be built in order to replicate the relationship with the researcher. Beyond the completion of the formal programme it was the lack of this social connection that led to individuals failing to continue with the exercise regime despite their awareness and understanding of its benefits.

While further research is required to assess the efficacy of these motivators in other population groups, these represent a clear guideline for exercise interventions in at-risk female populations.

## Conclusion

Post-menopausal females may initially be motivated to undertake exercise by the context of routine-based sessions in a supervised, small group setting of similar individuals. Education about health concerns is an important factor as this cohort desires a clearer understanding of the age- and hormone-related changes taking place in their bodies. Once the routine is entrenched the importance of social cohesion both with the researcher or group leader and the peers within the group is fundamental to retention. An emphasis on overall “wellbeing” is of greater value to this sample than considerations of aesthetics or weight loss.
